# Sociodemographic and clinical heterogeneity among longitudinal studies in dementia with Lewy bodies

**DOI:** 10.1002/alz.092858

**Published:** 2025-01-09

**Authors:** Joseph PM Kane, Federico Rodriguez‐Porcel, Ece Bayram, Carla Abdelnour, Philippe Desmarais, Isabella Delgado Echeverri, Manabu Ikeda, Maria Camila Gonzalez, Hideki Kanemoto, Kathleen L. Poston, Kathryn A Wyman‐Chick, Mario Ricciardi, Usman Saeed, Bedia Samancı, Yuto Satake, Yueyi Yu, Jin‐Tai Yu, James B Leverenz, Dag Aarsland, the ISTAART Lewy body dementias Consortia Working Group

**Affiliations:** ^1^ Queen’s University Belfast, Belfast, Northern Ireland United Kingdom; ^2^ Medical University of South Carolina, Charleston, SC USA; ^3^ University of California San Diego, La Jolla, CA USA; ^4^ Department of Neurology and Neurological Sciences Stanford University School of Medicine, Stanford, CA USA; ^5^ Centre hospitalier de l'Université de Montréal, Montréal, QC Canada; ^6^ Fundación Valle del Lili, Cali, Valle del Cauca Colombia; ^7^ Osaka University Graduate School of Medicine, Suita‐shi Japan; ^8^ Centre for Age‐Related Medicine, Stavanger University Hospital, Stavanger, Stavanger Norway; ^9^ Osaka University Graduate School of Medicine, Suita, Osaka Japan; ^10^ Stanford University, Stanford, CA USA; ^11^ HealthPartners/Park Nicollet Struthers Parkinson’s Center, Golden Valley, MN USA; ^12^ Ace Alzheimer Center Barcelona, Barcelona, Barcelona Spain; ^13^ Hurvitz Brain Sciences Program, Sunnybrook Research Institute, Toronto, ON Canada; ^14^ Istanbul Faculty of Medicine, Istanbul University, Istanbul Turkey; ^15^ Innovation Center for Neurological Disorders, Department of Neurology, Xuan Wu Hospital, Capital Medical University, Beijing China; ^16^ Huashan Hospital, Fudan University, Shanghai, Shanghai China; ^17^ Cleveland Clinic Lou Ruvo Center for Brain Health, Cleveland, OH USA; ^18^ Stavanger University Hospital, Stavanger Norway; ^19^ King’s College London, London, England United Kingdom

## Abstract

**Background:**

International collaboration is crucial to the future of research in dementia with Lewy bodies (DLB). Although it is hoped that a single global DLB cohort can be created through combination of data from the many longitudinal studies conducted internationally, a high likelihood of phenotypic heterogeneity may prohibit harmonisation and analysis of datasets. Our primary objective is to determine whether significant heterogeneity is observed in the sociodemographic and clinical characteristics of people with DLB globally.

**Method:**

Longitudinal DLB studies and multi‐centre consortia were invited through existing professional networks to contribute limited descriptive clinical and sociodemographic data for their samples using a standardised proforma. One way analysis of variance (ANOVA) with summary statistics as input was used to compare mean scores. χ^2^ test was used to compare categorical variables.

**Results:**

Descriptive data from 10 projects in seven countries, and one multinational consortium, were shared, comprising a pooled sample of 4157 individuals with DLB. The proportion of males (χ^2^ = 135, df = 7, *p*< .01) and the duration of formal education (*F*(3,6) = 19.41, p<.02) varied significantly between DLB projects. No significant difference in mean age (*F*(3,7) = 0.87, *p* = .61) or duration of DLB symptoms at baseline (*F*(2,6) = 1.46, *p* = 0.46) was noted, nor was it observed in scores of baseline Mini‐Mental State Examination (MMSE)(*F*(2,5) = 0.73, p = 0.66), Clinical Dementia Rating (CDR) (*F*(1,4) = 2.04, *p* = .48), or Part III of the Unified Parkinson’s Disease Rating Scale (UPDRS) (*F*(2,6) = 12.9, *p* = .73).

**Conclusion:**

Despite a broad range of project methodologies and a diverse group of subjects, little sociodemographic or clinical heterogeneity was observed between longitudinal DLB projects. Interrogation of subject‐level data, rather than descriptive data for each project, and more detailed clinical data on participants would allow further examination of heterogeneity. This could support harmonisation of individual DLB datasets to create a single global DLB cohort.

